# Europeanisation of health systems: a qualitative study of domestic actors in a small state

**DOI:** 10.1186/s12889-016-2909-0

**Published:** 2016-04-14

**Authors:** Natasha Azzopardi-Muscat, Kristine Sorensen, Christoph Aluttis, Roderick Pace, Helmut Brand

**Affiliations:** Department of Health Services Management, Faculty of Health Sciences, University of Malta, Msida, MSD 2080 Malta; Department of International Health, CAPHRI, Maastricht University, P.O. Box 616, 6200 MD Maastricht,, Duboisdomein 30, Maastricht, 6229 GT Netherlands; Institute of European Studies, University of Malta, Msida, MSD 2080 Malta

**Keywords:** Health policy, Health system reforms, European Union, Europeanisation, Qualitative study, Small states, Malta

## Abstract

**Background:**

Health systems are not considered to be significantly influenced by European Union (EU) policies given the subsidiarity principle. Yet, recent developments including the patients’ rights and cross-border directive (2011/24 EU), as well as measures taken following the financial crisis, appear to be increasing the EU’s influence on health systems. The aim of this study is to explore how health system Europeanisation is perceived by domestic stakeholders within a small state.

**Methods:**

A qualitative study was conducted in the Maltese health system using 33 semi-structured interviews. Inductive analysis was carried out with codes and themes being generated from the data.

**Results:**

EU membership brought significant public health reforms, transformation in the regulation of medicines and development of specialised training for doctors. Health services financing and delivery were primarily unaffected. Stakeholders positively perceived improvements to the policy-making process, networking opportunities and capacity building as important benefits. However, the administrative burden and the EU’s tendency to adopt a ‘one size fits all’ approach posed considerable challenges. The lack of power and visibility for health policy at the EU level is a major disappointment. A strong desire exists for the EU to exercise a more effective role in ensuring access to affordable medicines and preventing non-communicable diseases. However, the EU’s interference with core health system values is strongly resisted.

**Conclusions:**

Overall domestic stakeholders have a positive outlook regarding their health system Europeanisation experience. Whilst welcoming further policy developments at the EU level, they believe that improved consideration must be given to the specificities of small health systems.

## Background

The European Union (EU) acquired a health mandate in 1992 through the Maastricht Treaty, which is enshrined in Article 168 of the Treaty on the Functioning of the European Union (TFEU) [1]. This article must be read in conjunction with articles 3, 4 and 5 of the TFEU, which defines the competencies of the EU and the Member States as well as the so-called ‘mixed competences’. The essence of these Treaty provisions is that heath remains a Member State competence and Union action can only complement national policies but not supplant them. Therefore, in accordance with the principle of subsidiarity, the Union acts only insofar as the objectives of the proposed action cannot be sufficiently achieved by the Member States. Hence, the responsibility for organising and financing health systems remains a Member State responsibility in accordance with the principle of subsidiarity [2, 3]. Of course, this position leads to some ambiguity, which is also reflected in the mixed outcomes of European health policy [4, 5]. European level stakeholders perceive the results of the EU health policy as a mixture of achievements, failures and missed opportunities [6]. Whereas the EU has become a recognised player in the health sector, the extent to which it has actually made a difference to the health of European citizens is debated [7–9]. The EU has been described as exerting its influence on health systems through three main strands of activity: public health, market regulation and the European Semester [10, 11]. This situation has led to primarily ‘uninvited’ Europeanisation of health systems often resisted by domestic stakeholders [12]. The effects of austerity policies with the concomitant reduction in health budgets [13], particularly in Greece and Spain, have tended to generate a negative perception of EU action with regard to health systems [14–16]. An analysis of Country Specific Recommendations (CSRs) found that the European Semester^1^ system of fiscal and economic governance emphasises the financial sustainability of health systems over quality and accessibility [17]. Early analysis of the implementation of the patients’ rights and cross border care directive indicates the variable effects on Member State health systems [18–23]. These developments point to an increasingly important role for the EU in influencing health systems. In contrast, the Mission Letter issued by Juncker to the health commissioner in 2014 sends a clear message that the policy on health systems is best left to individual Member States [24]. Therefore, the future role of the EU regarding health systems appears unclear at this point, with either role expansion or retrenchment being possible outcomes.

The concept of Europeanisation has developed over time and has been largely defined as an outcome or a process [25].^2^ This paper adopts Radaelli’s definition of Europeanisation as ‘*a series of top-down and bottom-up processes affecting both formal and informal rules as well as procedures, policy paradigms, styles and shared beliefs and norms*’ [26]. This definition was selected because it includes policy instruments other than legislation and, therefore, is suitable for an assessment of health system Europeanisation considering that EU action in this policy area is often pursued through instruments other than legislation [27]. An examination of the domestic impact of the EU on the Maltese health system can be classified using the four types of outcomes that were described in the Europeanisation literature [28], namely: inertia, or Europeanisation occurring involuntarily if at all; retrenchment, or the continual resistance of EU pressures; adaptation, or making certain changes that do not affect the fundamentals of the system; and engaging in transformations that change the foundations of the domestic system, leading to paradigm shifts. The ‘goodness of fit’ hypothesis [29], although discredited as being too mechanistic and lacking empirical evidence in certain respects [25, 30], can still be usefully applied to an analysis of the Europeanisation of the Maltese health system. One expects that a high degree of misfit leads to transformation if domestic actors see value in adopting the European policy and actively utilizing the EU requirements as leverage to bring about change. Retrenchment or inertia would result where a conflict in values exists or the price tag associated with change is perceived to be too high from a domestic perspective. Adaptation or accommodation is likely to occur in situations where the degree of misfit is not unbearably high. Additionally, the literature on Europeanisation emphasises the importance of networks of elite stakeholders as mediating factors [31–33] in determining the overall effects of the EU on individual Member States. An investigation of domestic stakeholders’ viewpoints regarding the impact of the EU on their health system can be important in furthering our understanding of the manner in which the Europeanisation of health systems occurs. In addition, obtaining an understanding of domestic stakeholder expectations regarding the role that should be played by the EU in health systems can inform the manner in which health system Europeanisation occurs in the future.

Malta, which joined the EU in 2004, was selected as the setting for this study. An overview of the main features of the Maltese health system is provided in Table 1. Malta exhibits a relatively high level of engagement with the EU, as exemplified by the country’s positive track record in implementing legislation [34]. Malta is the smallest EU Member State and has no internal regional structures. A review of the effects of EU membership after a decade [35] carried out at the national level found that several sectors had been transformed, but also that the health sector does not receive any particular mention, which is consistent with the view that the health sector should be considered as a case of ‘least likely’ Europeanisation [33]. An examination of the impact of EU membership on the public service found that areas significantly affected by EU policy, such as customs and rural development, underwent significant policy change accompanied by structural and procedural reform. Areas less influenced by EU policy underwent little change. Following EU accession, two distinct types of public administration can be identified in Malta, namely that which is involved in EU policy and that which is unaffected by EU policy. That EU membership was a strong driver of public service change was concluded [34].

**Table 1:** Key facts about the Maltese health care system

Malta acceded to the EU in 2004. It is the smallest Member State in the EU with a population of 417,432 and a total land area of 315 km^2^. The publicly funded health care system is the key provider of health services. The private sector complements provision particularly in the area of primary care and ambulatory specialist care. The Ministry is responsible for setting policy and standards, for regulation of public and private health services as well as for funding and direct organisation and delivery of health care. The public health system is funded by general tax revenues. Total health expenditure was 8.7 % of Malta’s GDP in 2012 of which public spending was only 5.6 %. Sustainability of the health system has become identified as a key challenge and the Maltese health system has come under the scrutiny during the European Semester process. In 2013 and 2014 Malta has received Country Specific Recommendations (CSRs) calling for a comprehensive reform of the health system to improve the efficiency and sustainable use of available resources.	

Reproduced with permission from: [23]

Studies on the Europeanisation of health care were identified for cross-border mobility [36–38], health care coverage [39], alcohol policy [40], communicable disease policy [41, 42] and cancer [43]. However, to date no empirical studies were found that investigated the attitudes of domestic stakeholders towards health system Europeanisation.

In this paper, we apply the theory from European studies to explore the effect of the EU on health systems as experienced by domestic actors in the small EU Member State of Malta. Specifically, we assess how EU membership affected the Maltese health system. We also explore the attitudes of domestic health system stakeholders to the EU and seek their views on the future role they envisage for the EU with respect to health systems. We seek to fill an identified gap in the literature by going beyond an analysis of the manner in which the EU has influenced the Maltese health system and attempt to shed light on the normative dimension of health system Europeanisation [44].

## Methods

### Design

This qualitative study used information collected from face-to-face interviews to assess participants’ perceptions of the development of the Maltese health system within a European context. Permission to conduct this study was obtained from the University Research Ethics Committee at the University of Malta. The reporting of the study closely followed the COREQ criteria [45].

### Study participants and setting

Participants were recruited purposely with the principal inclusion criterion being the role they held in the health system or in European affairs over several years, such that they were already involved at the time of Malta’s accession to the European Union. Thereafter, they remained closely involved in decision making at the Malta–EU interface for a certain period. Therefore, the sample included senior public officers from the Ministries of Health and European Affairs, politicians, senior clinicians and leaders of civil society (Table 2). Thirty-five suitable participants were invited by the principal investigator and informed about the study. The voluntary nature of their participation was emphasised and precautions were taken to safeguard anonymity. Two persons declined to participate, citing lack of sufficient knowledge of the subject. Written informed consent was obtained from all participants before the start of every interview. All interviews were carried out only by the principal investigator and in English.^3^ Interviews were face-to-face in a location selected by the interviewee. Participants were informed that, through each interviewee’s personal unique experience, the scope of the interview was to contribute towards the construction of knowledge of how the Maltese health system was affected by EU membership. The interviews were audio recorded and transcribed verbatim.

**Table 2:** Professional roles of participants interviewed

Role	Number of participants
European affairs public officer	4
Ministry of Health (MoH) public officer	13
Politician	5
Academic	3
Clinician	3
Civil society	5
Total	33

### Interview guide

A semi-structured interview guide was developed from the literature on Europeanisation and small states and was reviewed by experts in public health, European studies and small state studies. Using this semi-structured interview approach ensured a fixed core of themes and allowed sufficient flexibility to digress and explore themes that emerged during the interviews. The themes in the interview included the following: participants’ experiences and views on the health policy-making process in Malta, examples of areas that changed as a result of EU membership, consequences for the health system associated with EU membership, the balance of competence between European and national policy making in the health sector, institutions and mechanisms through which the EU influences the health system and reflections on Malta’s size and implications for the policy-making process at the national and European levels. Further probing was carried out using supplementary questions primarily tailored to the background of the individual interviewee.

### Data analysis

An inductive approach was used to carry out the data analysis. Nvivo® 10 was used to support the coding process. To strengthen the validity of the data, the first five interviews were coded by three researchers to establish coherence and consistency. Subsequently, the remaining interviews were each coded by two researchers. The coding team consisted of the principal investigator and two researchers from Maastricht University distant to the Maltese health system. Additional codes were continuously added throughout the remainder of the analysis to preserve the richness of the data, bearing in mind that different stakeholders often emerged with unique perspectives. The list of codes that emerged was used to generate saturated clusters, categories and broader dimensions and themes.

Researchers compared their interpretation of the codes and divergences were discussed until consensus was achieved. The codes were grouped into categories from which key themes were identified. When determining the labelling of the codes and the categories, care was taken to preserve the original verbatim extracts of the study’s participants to ensure that their ‘voices’ remained visible throughout the research process [46]. Because the study aimed to highlight the normative dimension of the Europeanisation of the health system, this technique enhanced the authenticity of the data. Care was taken to emphasise the main points of consensus and convergence amongst the interviewees whilst also highlighting deviant views and unique contributions—where appropriate—to reflect the complexity that emerged from the stakeholder contributions [47].

## Results

**Fig. 1:**
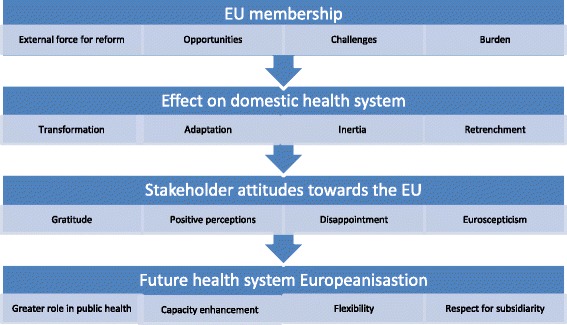
The process of health system Europeanisation in the Maltese health system

Thirty-three in-depth interviews were conducted with domestic actors during July and August 2014. The interviews were approximately 45 min and the total interviewing time was 23 h. Four major themes are identified and presented as a process, with each stage influencing the subsequent one (Fig. 1), in line with the research questions previously specified. The first theme represents the EU accession process and what EU membership signified in general terms. The second theme presents the specific effects of EU membership on the domestic health system. In the third theme, stakeholder attitudes towards the EU are depicted. The final theme presents stakeholder expectations regarding future health system Europeanisation. These four themes are analysed using supporting evidence from the data.

### General reflections on a decade of EU membership

The general consensus is that the net effect of EU membership has been *‘definitely’ (#25 politician)* and *‘undoubtedly’ (#27 MoH public officer)* beneficial, as illustrated through the following quote.

*‘Today’s citizen is more empowered; today’s citizen has more rights; today’s citizen in Malta benefits from much higher standards than he did or she did ten years ago, and that also holds in the area of health. So, whether it is the quality of the pharmaceuticals, the cross-border directive or freedom of movement and the level of specialization of the professionals who take care of the patients… I think there have been huge strides forward!’ (#18 politician)*

For public officers, the following important positive developments in the policy-making process are attributed to EU integration: a greater degree of transparency, a more structured consultation process, enhanced inter-sectoral cooperation and the requirement to consider the budgetary impact of the policies. Target setting is believed to have become more common, with an enhanced degree of accountability and a *‘better sense of discipline’ (#27 MoH public officer)* also characterising the policy-making process.

The majority of interviewees believe that the obligation to comply with EU rules was important for certain sectors in Malta to *‘evolve’* (*#7 MoH public officer)*. Stakeholders describe EU membership as *‘being part of a family’ (#3 politician)* or *‘a community which has its eyes on us’ (#1 academic)*. This external scrutiny is deemed important to introduce norms and appropriate behaviour for policy makers. This benefit of having *‘checks and balances’* (*#*3 *politician)* in the policy-making process is described as a significant impact of Malta’s EU membership.

*‘Securing independence is one thing, but setting up the institutions, introducing checks and balances is most essential and I think, perhaps, this is the gift of the European Union to us’ (#4 clinician).*

The external pressure brought to bear by the EU assisted politicians to overcome barriers and ‘*face politically difficult decisions’ (#18 politician)*, often forcing them to do things they would not have done because of competing priorities. The accession process is described as a *‘golden period’ (#1 academic)* and a ‘*catalyst’ (#29 MoH public officer),* bringing about rapid changes and the establishment of standards.

*‘What the European Union has helped us to do is to actually achieve a lot in a very short period of time, and thankfully, it was that way because otherwise we would probably have not succeeded’ (#11 civil society)*.

However, the accession process also brought enormous challenges for the small administration, and a public officer described the situation as, ‘*really swimming against the current’ (#13 MoH public officer)*. A lack of capacity to meet EU requirements (or norms) and the administrative burden associated with excessive bureaucracy are two main challenges that most interviewees identified. The administrative burden is the ‘*price to pay’ (****#****18 politician)* for becoming a member of the EU and a key challenge is the shock of having to adapt from being an organisation with an absent documentation culture to becoming part of a system in which documentation plays a key role. This increased workload and burden associated with answering questionnaires or attending meetings is negatively perceived as taking up valuable time that could be better spent working on the core public health business. This lack of capacity to keep up with EU demands also affects civil society.

*‘It is hard to keep up with the changes because when you feel that you have come home with the transposition of one EU directive, there will be other directives in the making, opinions, positions, green papers, papers of all the colours under the rainbow’ (****#****15 civil society).*

### Effect of EU membership on the domestic health system

The reform of the pharmaceutical sector and the development of regulations for specialist health care professional qualifications are believed to be the most important health system domains that were transformed as a result of EU membership. EU membership provided an opportunity to overhaul and modernise the legislative framework for public health regulation, including communicable disease control, food safety and environmental health, which were all significantly strengthened through institution building. These sectors are all subject to comprehensive legislation at the EU level that had to be transposed and implemented in the domestic health system.

*‘There are three or four sectors which were completely revolutionised since we joined [the EU] medicines, healthcare professionals or rather how they are regulated, food safety, public health issues…’ (#10 MoH public officer).*

Some stakeholders point out the important impact on specific services and mentioned ‘*cancer screening services’ (#21 MoH public officer)* and *‘major, major improvement in the blood transfusion services’ (#20 civil society).* These areas were also the subject of an EU recommendation and directive, respectively. However, stakeholders have mixed feelings about the effectiveness of non-binding recommendations as effective mechanisms for implementing change because–in the area of patient safety–insufficient improvement is considered to have occurred. In line with this observation, whilst interviewees hold divergent views regarding the extent to which EU integration affected the actual organisation of health services, the overall perception is that health system financing and delivery has mostly been unaffected.

*‘I don’t’ think it has made much of a difference really. Healthcare as such hasn’t changed-the actual provision of healthcare, the quality, the timing or the delivery of the service–I think all that hasn’t changed at all’ (#23 clinician).*

The lack of a legislative EU obligation in certain health services is considered a missed opportunity for introducing a much-needed reform. In primary care, no significant reforms were implemented other than the specialist training programmes for general practitioners, which is a mandatory EU requirement. An ambitious reform proposal for primary care failed after encountering stiff stakeholder resistance. For some interviewees, this reform failure in primary care is partly attributed to the lack of an EU-driven obligation.

*‘I think if there was some sort of directive, or recommendation, or opinion, or strong push from Europe, it would help us to push things forward in primary care’ (****#****2 MoH public officer).*

However, public officers do not view the EU influence as being limited to areas in which a legislative obligation exists. They perceive a more indirect and ubiquitous influence on various aspects of the health policy-making process itself, with the formulation of public health strategies in areas such as sexual health and non- communicable diseases being attributed to indirect influence of the EU. The number of health strategies launched is reported to have increased markedly following EU membership.^4^ Public officers feel that they were *‘pushed’ (#17 MoH public officer)* by the EU to develop a national health strategy. They describe how the need for such a strategy has long been identified by the public health community but was only accepted as a priority by the political class when it became a conditionality to access European funding.^5^ Other specific benefits for the health system resulted from the use of EU funds. Constructing and equipping an oncology hospital and the training and development of health professionals are important examples of health system development and service transformations that were made possible through EU funding.

Not all effects of the EU on the domestic health system are positively regarded. Compliance with the working time directive is a major health system challenge through which stakeholders believe that the EU demonstrates a lack of understanding of the specificities associated with running a small health system. Participants expressed their concern that removing the opt-out clause that allows workers to exceed 48 h weekly would mean that ‘*health services would collapse’ (#20 civil society).*

A second major health system challenge identified refers to the reform of the pharmaceutical sector. Although interviewees acknowledge the benefits of increased consumer protection as a result of the implementation of EU law on the quality, safety and efficacy of medicines, they describe serious concerns about the decrease in the availability of medicines in the market and price increases following EU accession. Stakeholders question whether the regulatory regime adopted was *‘too draconian’ (#18 politician) f*or such a small health system and whether a more efficient system could have been considered.

Stakeholders expressed mixed views about the cross-border directive. Although it is too early to judge the overall effect, some believe that the directive has unrealistically raised patients’ expectations, thereby posing a potentially serious challenge for the health system. Others play down its significance.

A public officer knowledgeable about the EU fiscal governance regime registered her grave concern about the increased EU focus on the financial sustainability of health systems. She questioned whether domestic policy makers would be able to continue to resist pressures to implement changes to the health services system provided free of charge at the point of use.

*‘From the financial point of view, from the budgetary point of view, the EU is focusing more on the sustainability of our health system, and I think that will be the major challenge in the years to come’ (#33 EU Affairs public officer).*

During the first decade of EU membership, the Maltese health system has undergone several changes through an array of EU mechanisms. Public health and those sectors for which the EU has legislative competence through its internal market legislation were particularly affected. However, core health system elements, including the financing and organising of services, remained largely unchanged (Tables 3 and 4).

**Table 3:** Health system transformation and adaptation (Malta 2004–2014)

Health system change			
Description	Europeanisation	Mechanisms	Analysis
Public health policies and strategies	Transformation	Non-binding EU communications, strategies, reportsParticipation in EU working groups	Domestic health policy-making process underwent significant change and a number of important health strategies were developed
Cancer	Transformation	Non-binding EU Council Recommendation on Cancer Screening EU Funds for hospital, equipment and capacity building Participation in EU Joint Actions and networks Submission of health information statistics	Services in the area of cancer have been transformed through the development of a national plan, cancer screening services, training of health professionals and the constructions of a new oncology hospital
Development of specialist training programmes for doctors	Transformation	Directive EU funds for capacity building	Transposition of legislation and establishment of medical specialist registers as well as structured post graduate training programmes
Regulation of quality, safety and efficacy of medicines	Transformation	Directives EU funds for capacity building Participation in networks and working groups	Transposition of legislation and setting up of the competent authority to regulate the placing of medicines on the market
Establishment of regulatory institutions with separation of regulatory and provider roles	Adaptation	Directives Participation in networks and working groups	Transposition of legislation and setting up of competent authorities for licensing providers and regulating public health standards
Health statistics	Adaptation	Participation in networks and working groups Benchmarking (EUROSTAT regulations recently entered into force)	A good health information system was already in place prior to accession but EU legislation, policy and networking helped to strengthen it

**Table 4:** Inertia and resistance to health system reform (Malta 2004–2014)

Health system continuity			
Description	Europeanisation	Mechanism	Analysis
Primary care	Inertia	Directive (on training of general practitioners)	The necessary changes were implemented to the specialist training for general practitioners but otherwise no significant changes were reported and the planned 2009 reform was not implemented
Patient safety	Inertia	Non-binding EU Council Recommendation on Patient Safety	Reports on the implementation of patient safety indicate that the Maltese health system has not made any significant advances on this aspect
Cross border care	Inertia	Directive	Transposition of minimal requirements of the directive
Pricing and reimbursement	Inertia	Directive	Minimum requirements of the transparency directive on medicines were transposed but no major changes to the system of pricing or reimbursement were implemented
Working time	Retrenchment	Directive	Extensive use of the ‘opt-out’ clause for doctors agreeing to work more than 48 h weekly so as to avoid major changes to the system
Funding of public health care	Retrenchment	Country specific recommendations emerging from EU fiscal and economic governance mechanisms	Despite health system sustainability being repeatedly mentioned in several annual reports the model of health financing has been strongly protected by successive Governments

A minority of stakeholders are of the opinion that the changes observed in the Maltese health system would have happened anyway, but that EU integration hastened the implementation of the reforms. A small number of stakeholders questioned whether the role of the EU in influencing health system development is overstated and suggested alternative explanations, such as national political priorities, globalisation, a neoliberal agenda, access to information from the Internet and the role of the World Health Organisation, as other important drivers for health system reform.

### Domestic health system stakeholder attitudes towards EU integration

A range of domestic health system attitudes towards EU integration are identified with the overall perception being a positive one despite the burden connected with a disproportionate bureaucracy on the small administration.

*‘Thank God that there is an obligation, so thank God for the EU!’ (#21 EU affairs public officer).*

This extract captures the sense of gratitude that several stakeholders associate with EU integration. Positive attitudes towards EU integration stem from various perceived benefits. Access to knowledge and information obtained from other MSs, which avoids the need to *‘reinvent the wheel’ (#1 academic)* and the concept of *‘riding the EU bandwagon’* (*#7 MoH* health public officer) in carrying out joint assessment work in medicines are important examples of mechanisms that alleviate the administrative burden. Capacity building and European peer networking are viewed as instrumental to overcoming the loneliness and professional isolation associated with working in policy and regulation in a small island. EU membership has facilitated international networking at the clinical level, which indirectly serves to raise healthcare standards and improve morale and self-confidence amongst clinicians. Networking is also described by civil society representatives seeking to promote their members’ interests at the EU level as having increased in importance. In addition to networking, the importance of access to technical assistance and specialised expertise is highly valued. The European Centre for Disease Control (ECDC) is an important source of support for the domestic public health workforce.

*‘ECDC has given us very important support. For example, it brings a group of experts together and they develop guidelines. So, for us, that is very good because we don’t have such a wide pool of expertise. We have 24-hour communication with ECDC and it is not the first time that they carried out assessments, even specifically for us. Last year we had Q fever. It was the first case that we actually came across in the last few years and we wanted guidance. ECDC actually carried out an assessment for us. We have reassurance that we have someone to turn to’ (#6 MoH public officer).*

However, despite these positive examples, several stakeholders believe that *‘The approach of the EU to health …is disappointing’ (#6 MoH public officer).* Disappointment is a result of the lack of priority accorded to health at the EU level. Health ministers are viewed as being weak in relation to their finance counterparts. Poor budgetary allocations and the limited power of the Commission Directorate General responsible for health are viewed as resulting in a weak stand when confronting multi-national lobbies. The tobacco and food industries raise a particular concern. Another key source of disappointment is the *‘one size fits all’* approach, which belies a lack of understanding of the specific challenges faced by MSs given their geo-demographic or socio-economic profiles.

*‘Most of the people who are taking the decisions in Brussels come from large countries and they may not perceive what our problems are. For example, one maternal death is sufficient to screw up your data…’ (#26 clinician).*

Specifically, public officers and academics expressed their disappointment and frustration at being unable to tap into EU funds to develop local research capacity, with EU funds invariably going to centres in larger countries in which cutting-edge research is taking place. A politician who expressed his belief that *‘there are funds provided you apply in a diligent way and abide by the rules’ (#19 politician)* dissented starkly from the general consensus. The co-funding element, lack of capacity and administrative bureaucracy are all listed as key barriers to accessing EU research funds. Small states’ particular needs are also believed to be often overlooked in impact assessments. Whilst a couple of initiatives to lighten the burden exist in the pharmaceutical sector, they are deemed to fall far short of addressing small state specificities and are viewed as providing an exceptional *‘way out instead of having an infrastructure which is friendly to small member states’ (#31 EU affairs public officer)*.

An interviewee with extensive experience in technical meetings uniquely stated that his requests about small size issues *‘are typically then taken on board, although to varying extents’ (#17 MoH public officer)*, thereby illustrating the importance of intervening early in the initial technical stages to maximise influence.

Disappointment is also related to a series of unmet expectations, foremost amongst which is the lack of EU engagement and support related to the problems posed by immigration.

*‘The biggest disappointment, not just in healthcare but for the whole Maltese population although it also is important for healthcare, is the failure of the EU to engage with immigration’ (#4 clinician).*

The gap between rhetoric and documentation produced at the EU level and tangible change at the operational level, lack of continuity between EU Presidencies and inability to ensure effective enforcement are other examples of work being carried out at the EU level but that is not making the desired impact at the domestic level.

*‘Decisions are taken at the very top by the ministers but the problem mainly is whether they seep down and are actually implemented at the operational level. There is a huge gap’ (#30 MoH public officer).*

Amongst some public servants, disappointment associated with unmet expectations coupled with fatigue from the struggle to cope with daily EU pressures appears to be leading to Euroscepticism.

### Expectations regarding future health system Europeanisation

The role that the EU should play in public health policy and health systems is contentious. Some stakeholders state that *‘people want subsidiarity to stay, they want to run their own system’ (****#****20 civil society), whereas* others express a desire for a greater degree of EU involvement. Even those participants who agree with a greater role for the EU acknowledge the sensitivity that exists around the principle of subsidiarity and the problem of the existing diversity of European health systems that prevent the EU from assuming a larger role.

*‘Let’s imagine that reforms in primary healthcare will be driven at the EU level across Europe, let’s dream about that! But then again you cannot really have the ‘one size fits all’ because that would result in chaos because really, you can’t standardize practices like that’ (****#****1 academic).*

Growing Euroscepticism and the increasing fiscal and economic orientation being taken by EU institutions are believed to be contributing to heightened tension between Europeanists and pro-autonomy forces.

*‘There are two schools of thought, those who think that the European Union should exercise greater control possibly from Brussels versus those who want more space for Member States to decide for themselves. Although these divisions have always existed in the European Union, I think that they will now become more prevalent’ (****#****24 MoH public officer).*

The following phrase captures the general feeling amongst most interviewees: *‘it would be more beneficial that Member States are actually doing more together rather than the opposite’ (****#****8 EU Affairs public officer). S*everal policy areas in which a greater role for the EU is deemed both desirable and feasible are identified. Foremost amongst these areas is the issue of access to affordable medicines. Since the financial crisis, the issue of affordability of medicines and attention to pricing has been noted to be no longer only of concern to small or poor countries but also has affected MSs, which was hitherto unaffected by it. Action at the EU level can counteract the limitations associated with a small market size.

*‘Most Member States are now facing sustainability and pricing issues, so I guess, the EU through better cooperation, could help them face these challenges jointly’ (****#****31 EU affairs public officer).*

Several interviewees feel that the EU should play a more active role in the prevention and control of non-communicable disease, with obesity, diabetes and tobacco control considered as key priorities.

*‘If I had to pinpoint one area where the EU could come together more effectively is in the major non-communicable diseases to make sure that what is being done at national level in twenty-eight different countries, is shared, brought together and supplemented at EU level to make sure that we get the best results faster and translated into more effective remedies that can be shared by all patients affected across the EU’ (****#****18 politician).*

The introduction of a basic level of care and a standard health care package across the EU, as well as standards for primary care, are considered important future developments for EU health policy by domestic stakeholders. The adoption of minimum standards of training and qualifications for specialist nurses, for allied health care professionals and for carers is considered a priority. A few interviewees expressed their desire for the EU to play a larger role in quality and patient safety. Some interviewees see the need for the EU to take a more active role in developing health information systems. Voluntary mechanisms, such as using enhanced cooperation procedures, are proposed as methods to implement such measures to ensure flexibility and avoid the much maligned ‘one size fits all’ approach.

In keeping with the general desire for the EU to play a larger and more visible role, most interviewees do not believe that excessive involvement or undue influence of the EU exists on the Maltese health system. The notable exception is the perceived EU influence on curbing public health care expenditures, which is unequivocally deemed as a threat by all stakeholder groups. The EU’s pressure to curb health sector expenditures is believed to reflect an insufficient understanding of the domestic health system context and that negative consequences for the health system could result from such approaches. A public officer strongly expressed his view that it is very important to defend the principle of retention of a health service that is free of charge at the point of use.

*‘My feeling was that the Commission was trying to exert a bit too much influence and the worst thing about it was that the people making those suggestions or making those statements were coming from economical background. So, if I may daresay, their recommendation does not only belie certain ignorance of the local context, but also of basic public healthcare principles’ (#17 MoH public officer).*

Although some clinicians feel that the EU should play a role in setting down basic care standards, others hold that this role should remain within the remit of scientific bodies and that European institutions should not attempt to replace scientific guidelines with bureaucratic ones. Regarding human resource planning and deployment, public officers and civil society representatives hold divergent views on the extent to which national control on decision making should be retained; however, one participant described mandatory staff patient ratios at the EU level as a *‘no-go situation’ (#13 MoH public officer)*.

Interviewees recognise the need for the EU to coordinate between the different MSs and acknowledge the support provided, particularly by ECDC in the area of communicable disease control. However, a detailed approach should be elaborated on at the MS level and the EU should refrain from taking up roles that are already suitably catered for by other organisations, such as the World Health Organisation (WHO).

*‘I think that the Commission is there to coordinate what happens across EU but then it is up to individual Member States to manage their response because each Member State has different capacities, different limitations and different cultures’ (#6 MoH official).*

Finally, religiously inspired values, including issues concerning reproductive health and abortion in particular, are an important unique theme for Maltese stakeholders. Any attempt by the EU to set policy would be strongly resisted.

## Discussion

### Summary of key findings

Figure 1 illustrates how the process of health system Europeanisation is perceived to have occurred in Malta. The accession process provided a unique opportunity for health system reform, particularly in the area of medicines and professional training. However, other aspects of the health system, including the mechanisms of financing and delivery, were unaffected. Stakeholders positively view the EU as offering important support through technical and financial assistance and capacity building as well as in overcoming local sources of resistance to change. Negative attitudes are associated with administrative burdens and conflicting values. Overall, domestic stakeholders in the Maltese health system are positive over the EU influence on their health system and desire greater EU involvement in health policy as long as the influence is flexible enough to take into account small state specificities.

### Health system Europeanisation in practice

Tables 3 and 4 show how the degree of Europeanisation within the Maltese health system has varied amongst the different health system domains. Where Europeanisation has occurred, it has been done through diverse mechanisms–confirming that both regulatory compliance and social learning play a role in the Europeanisation process [44]. The window of opportunity to implement reforms provided by the EU accession process and described in this study confirms the findings from the literature in other sectors [48–50]. The highest adaptation pressures were experienced in the pharmaceutical sector and mutual recognition of professional qualifications, including medical specialist training. This finding is not surprising given that the principle of free movement underpinning these sectors is a foundational EU policy [51–53] and both areas were highlighted as being impacted in pre-accession assessments of candidate countries [54], including Malta [55, 56]. The impact on the pharmaceutical sector in Malta was also previously described [57]. Malta did not experience public health reforms associated with accession on the same scale as that reported in other countries [58], and the health services’ core elements appear to have remained mostly unaffected. For some stakeholders, this phenomenon represented a missed opportunity to bring about change and is most evident in the area of primary care. In primary care, a series of proposals for reform failed to materialise [59], and stakeholders appear to believe that an EU obligation would most likely have provided the necessary impetus for reforms to be implemented.

Therefore, this study established that, to date, the dominant focus for health services organisation and delivery resides at the national level. However, Hervey’s observation that the influence of the EU permeates *‘virtually every aspect of such [health] policies’* [5] also receives support from our findings because the EU appears to be exerting an indirect effect on health policy making by stimulating the production of several national health strategies.

A manifest implementation gap between what is decided at the EU level and the effect within the health system emerged as an important critique of the effectiveness of Europeanisation in practice. This consideration is important because health policy is governed to a large extent through soft law, which–although considered to play an important role [27, 60]–our findings indicate has mixed effects. For example, stakeholders describe the implementation of the recommendation on cancer screening as a success but the implementation of the recommendation on patient safety as poor. The effectiveness of implementation has been found to vary among countries [61], and small states must often prioritise because of their limited capacity [62, 63]. In these circumstances is it not surprising that non-mandatory initiatives assume a lower priority. Furthermore, the existence of strong veto players is likely to affect the ability of governments to implement non-binding recommendations.

### A small state perspective on health system Europeanisation

A survey of European health stakeholders found mixed perceptions of whether or not role expansion should occur for the EU in health policy [6]. A study carried out in the United Kingdom on the balance of competence between the EU and MSs in the field of health policy concluded that the balance is ‘broadly right’ [64]. Stakeholders from our study in the Maltese setting demonstrate support for further EU involvement in certain areas in which action at the level of a small state is deemed insufficient to achieve the desirable public health results. Therefore, the expansionist stakeholder attitudes towards future EU health policy observed in this study can partly be explained as being a result of the special characteristics of small states. Small states benefit disproportionately from the existence of effective regional organisations [65] and ‘soft’ security aspects, including public health, have been described amongst such benefits [66]. For example, the literature on the value of ECDC is mixed. Some hail this institution as a policy success [6], whereas others question its ability to fulfil its mission because of its heavy reliance on country experts [7, 67]. Our study found that Maltese health system stakeholders are strongly positive about the role played by the ECDC. Therefore, discussions on the future role for ECDC should also consider the benefits that accrue to small MSs. Networking as a means of overcoming professional isolation emerged as a substantial benefit for domestic stakeholders. Furthermore, the emerging global interdependence of public health [68] makes it even more pressing for small states to acquire the protection and shelter of a regional organisation to defend public health interests [66]. This study revealed a desire for the EU to play a larger role in ensuring access to affordable medicines, a key issue for the small domestic market. The Joint Procurement Agreement on medical countermeasures for cross border health threats [69] and the setting up of an expert working group on safe and timely access to medicines [70] are examples of policy initiatives that have been championed by small states.

However, the desire for a larger EU role in health systems is offset by stakeholder disappointment with the lack of understanding of specificities related to the geo-demographic profile of Malta. Although the literature has traditionally portrayed the Commission as being an ally for small states [62, 71], our study found that this portrayal is not always the case. A potential explanation is that many key decision makers in EU institutions hail from larger countries. The ‘one size fits all’ approach appears to have created problems in the implementation of the working time directive, aspects of the pharmaceutical Acquis [72] and in access to research funding. The lack of public health research in small states has been described elsewhere [73–76] and the findings from this study serve to confirm that this lack of research remains a particular challenge for small states.

### Critique of Europeanisation theory

The typical dilemma of establishing causality in Europeanisation research emerges in this study [77]. One may question whether the role of EU integration as a catalyst for reform is overstated and whether change could equally have resulted from other influences [78]. The broad consensus amongst stakeholders interviewed is that beyond the necessity of regulatory compliance–markedly associated with the accession process–the overall on-going change attributed to EU influence within the health system is brought about primarily through networking. This consensus concurs with the concept of socialisation and social learning as vehicles for Europeanisation as described in the social constructivist model [29, 44]. In these circumstances in which no EU regulation or directive exists as a point of reference, it becomes far more difficult to determine how much of the observed change is driven by the EU as opposed to other forces resulting from economic globalisation or neoliberalism.

### Strengths and limitations

This study is innovative and attempts to cover a broad scope. Efforts were undertaken to ensure reflexivity through the research process [47]. The core research team consisted of three individuals, two PhD students with previous public health research experience and one post-doc researcher with public health practice and experience in qualitative research methodologies. The principal investigator is based in the Maltese health system and the collaborating researchers are in The Netherlands. Their different locations allowed in-depth contextual knowledge to be complemented by external assessment and provided a forum for reflecting on the study design and analysis, and to critically question the process at all stages. The principal investigator previously occupied senior positions within the Ministry of Health in Malta, including responsibility for European and international affairs. The motivation for this research stemmed from an interest in investigating the impact of EU membership on the health system in Malta. All participants were recruited through the professional network of the principal investigator who did not share her own opinion until the interview was complete, even when this opinion was requested by the interviewee because the perception of the interviewee was the main focus of the interview [79]. Despite all of the steps taken to assure quality, this study has certain limitations. This study provides a picture of the situation through the lens of domestic stakeholders at a single point in time and focuses particularly on a number of issues related to Malta. Thus, the findings may not necessarily transfer to other contexts, and further research is necessary to determine whether other small countries face similar challenges. Additionally, complementary approaches using different techniques, such as process tracing, may be performed in the future to validate the findings and to strive to overcome the limitations previously described in establishing causality. Nevertheless, this study contributes important innovative perspectives on European health policy, and further research amongst domestic stakeholders in other Member States is recommended.

## Conclusions

Establishing causality is a dilemma for researchers in the field of Europeanisation. Yet, the findings from this study appear sufficiently strong to indicate that domestic stakeholders believe that Malta’s integration into the EU provided an external drive for certain reforms to be implemented. Public health policies appear to be affected more by EU policy than health care services. A policy infrastructure that is ‘friendly’ to small Member States is deemed preferable to the creation of specific exceptions. We found evidence of both ‘passive downloading’ of EU regulations and ‘active usage’ of EU rules to promote the desired norms and objectives. Although the health sector is a peripheral policy area for the EU, merit exists in using Europeanisation as a concept to better understand the evolution of this policy area in the EU. Obtaining a deeper understanding of the interaction between the EU institutions and MSs and the tension between, on the one hand, the desire for a larger EU mandate and, on the other hand, the safeguarding of subsidiarity is critical. This understanding is particularly relevant in view of the current context in which health systems are being increasingly framed in terms of financial and economic considerations with the potential marginalisation of public health from the policy objectives at the EU level.

We conclude that domestic health system actors in Malta generally share a positive assessment of the overall impact of EU membership on the health system and support a larger role for the EU in several policy areas. This support is generated from positive experiences, from a sense of disappointment that not enough is being done at the EU level to promote public health and from a desire that the EU provides support to overcome domestic health system problems linked to small market size. At the EU level, the financial crisis and ensuing effects on several health systems may provide an important opportunity to alter the propensity of at least some Member States to engage in more intensive health system cooperation. This study, by providing a small state perspective to health system Europeanisation, challenges the traditional narrative that Member States do not see a need for deeper integration in the field of health policy. What would be interesting to establish in this context is whether this need for deeper integration is felt by all states or whether it is felt more intensely by smaller states that lack sufficient resources, knowledge and policy initiatives but benefit from uploading their problems to the EU level or finding additional resources that they individually lack. Therefore, this study sets the scene for broadening the analysis to other small states to ascertain whether our findings are uniquely applicable to Malta or to small EU member states in general. However, other interesting possibilities exist that arise from our study, including the question: do larger states face similar challenges and dilemmas in their health systems at regional and local levels and would our findings and arguments apply equally to them?

## Footnotes

1 The European Semester is an EU-level policy co-ordination tool that contributes towards the broader EU aims of strengthening economic governance and greater policy co-ordination. This tool provides a more integrated surveillance framework for the implementation of fiscal policies under the Stability and Growth Pact, and the implementation of structural reforms through national reform programmes. The Commission publishes Country-Specific Recommendations for each Member State on the basis of a thorough assessment of every Member State’s plans for sound public finances and policy measures to increase growth and jobs. For further information, the reader is referred to http://ec.europa.eu/europe2020/making-it-happen/index_en.htm [accessed on 6 September 2015]

2 For a comprehensive yet simple introduction to Europeanisation theories, the reader is referred to Harwood Mark, Chapter 3 Europeanisation in Malta in the European Union Ashgate Publishing 2014.

3 English is an official language in the Republic of Malta.

4 The main health strategies published during the period following accession are listed in Chapter 6 of the Health Care Systems in Transition Report for Malta, which is available at http://www.euro.who.int/en/about-us/partners/observatory/publications/health-system-reviews-hits/full-list-of-country-hits/malta-hit-2014 [accessed on 09/10/15].

5 The European Commission issued guidance on a number of conditionalities that need to be fulfilled for access to the European Structural and Investment Funds 2014–2020. In the area of health, the existence of a strategic plan for the health system is one such criterion. Further information is available at http://ec.europa.eu/regional_policy/index.cfm/en/information/legislation/guidance/ [accessed on 6 September 2015].
